# Identification of key genes in salivary gland in Sjögren’s syndrome complicated with Hashimoto thyroiditis: Common pathogenesis and potential diagnostic markers

**DOI:** 10.1097/MD.0000000000035188

**Published:** 2023-09-29

**Authors:** Kaiyuan Zhang, Xue Yu, Yuxin Zhang, Dingqi Lu, Xinyi Yao, Tao Hong, Yating Ren, Liying Chen, Xinchang Wang

**Affiliations:** a Second Clinical Medical College, Zhejiang Chinese Medical University, Hangzhou, Zhejiang Province, People’s Republic of China; b First Clinical Medical College, Zhejiang Chinese Medical University, Hangzhou, Zhejiang Province, People’s Republic of China; c Department of Rheumatology, The Second Affiliated Hospital, Zhejiang Chinese Medical University, Hangzhou, Zhejiang Province, People’s Republic of China.

**Keywords:** complications, diagnostic model, Hashimoto thyroiditis, hub genes, Sjögren’s syndrome

## Abstract

The coexistence of Sjögren’s syndrome (SS) and Hashimoto thyroiditis (HT) has been confirmed, but the common mechanism of its co-occurrence remains unknown. This study aims to further explore the underlying mechanism and biomarkers for the co-occurrence of SS and HT. The Gene Expression Omnibus databases were used to obtain gene expression profiles for SS (GSE127952 and GSE23117) and HT (GSE29315 and GSE138198). Following identifying SS and HT’s shared differentially expressed genes, functional annotation, protein–protein interaction network creation, and module assembly were performed to discover hub genes. H&E staining and immunohistochemistry were performed to validate the expression of the hub genes in salivary glands. Finally, the receiver operating characteristic (ROC) curve was utilized to assess the discrimination of the hub genes as biomarkers in predicting SS, this study applied CIBERSORTx to analyze the immune infiltration in SS and HT in addition. A total of 48 common differentially expressed genes (48 upregulated genes and 0 downregulated genes) were chosen for further investigation. We analyzed the expression and function of PTPRC, CD69, IKZF1, and lymphocyte cytosolic protein 2 via H&E, immunohistochemistry, and ROC analysis. The 4 hub genes were mainly enriched in the T-cell receptor signaling pathway. We then evaluated and verified the diagnosis value of 4 hub genes in clinical minor labial gland biopsy of SS with HT, SS without HT, and non-SS. ROC analysis revealed that the 4 hub genes had a strong diagnostic value. Our study showed the common pathogenesis of SS and HT. These hub genes and diagnostic models may put forward some new insights for diagnosing and treating SS complicated with HT.

## 1. Introduction

Sjögren’s syndrome (SS) is a typical systemic autoimmune illness by salivary glands and lacrimal glands focal lymphocytic infiltration, resulting in dryness of the mouth and eyes.^[1]^ The etiology and pathogenesis of SS are complicated, in which autoimmune responses based on genetic susceptibility, epigenetics, and environmental risk factors play a crucial role.

HT is characterized by the presence of thyroid-specific autoantibodies and is one of the most common autoimmune diseases. Due to the autoimmune destruction of this gland, a gradual thyroid insufficiency in affected patients. HT is characterized by an immune response of Th1 cells, where the attack of T cells on the thyroid gland leads to an inflammatory response and overexposure of thyroid antigens, resulting in the secondary production of thyroid peroxidase antibody(TG-Ab) and thyroglobulin antibody (TPO-Ab).^[2]^ Current studies have confirmed that primary Sjogren syndrome (pSS) can be associated with organ-specific autoimmune diseases and complicated by systemic involvement, such as primary biliary cholangitis, autoimmune thyroiditis, pulmonary fibrosis, and so on.^[3]^ A retrospective study of 467 patients with pSS found that the prevalence of Hashimoto thyroiditis (HT) in patients with pSS was about 6.28%, while the prevalence of HT in the general population was only 1 to 2%, which is much lower than that in patients with SS.^[4]^ Based on this, we speculate that pSS patients may have some predisposing factors that make them more susceptible to HT.

Although the exact causes of SS and HT have not been fully clarified, SS and autoimmune thyroid diseases represented by HT have many genetic and immunopathological similarities and have some common characteristics.^[5]^ For example, Both SS and HT are associated with the same environmental factors including viral infections, cigarette smoking, alcohol intake, and stress.^[6,7]^ Both labial glands and thyroid tissues under pathological conditions have lymphocyte infiltration, especially CD4+T lymphocytes.^[8]^

Based on the histological function correlation between SS and HT and the similarities in environmental factors and pathogenesis, we surmised that there may be common genetic susceptibility factors and pathogenic pathways. Therefore, we analyzed the gene expression databases of 2 salivary glands and 2 thyroid glands via bioinformatics analysis, and further confirmed the coexpression of key genes in SS and HT. Then, we investigated the underlying mechanism of these genes and confirmed their expression in salivary gland biopsy samples. Finally, the receiver operating characteristic (ROC) curve was used to evaluate the veracity and reliability of the hub markers in diagnosing SS. The hub genes and diagnostic model identified between SS and HT are expected to provide a new understanding of the underlying mechanisms of the co-occurrence of SS and HT, and help to formulate early diagnostic strategies, prognostic markers and therapeutic targets.

## 2. Materials and methods

### 2.1. Gene expression omnibus datasets and the identification of differentially expressed genes

The thyroid glands and salivary glands gene expression profiles of GSE29315 (n = 6 HT, n = 8 control), GSE138198 (n = 12 HT, n = 3 control), GSE127952 (n = 8 SS, n = 6 control), and GSE23117 (n = 11 SS, n = 4 control) were collected from Gene Expression Omnibus (GEO) (https://www.ncbi.nlm.nih.gov/). We used GEO2R online software (http://www.ncbi.nlm.nih.gov/geo/geo2r) to analyze GSE29315, GSE138198, GSE127952, and GSE23117 databases and identified the differentially expressed genes (DEGs). A *P* value <.05 and log | FC |  ≥1 were set as the cutoff criteria for DEGs. Genes with a log | FC | ≥1 were regarded as upregulated genes, and those with a log | FC | ≤–1 were regarded as downregulated genes.

### 2.2. DEGs enrichment analyses

To further illustrate the function of DEGs, gene ontology (GO), and Kyoto Encyclopedia of Genes and Genomes (KEGG) pathways analyses were performed using the DAVID database (https://david.ncifcrf.gov/) and ClueGO^[9]^ in Cytoscape 3.7.1.

### 2.3. Integration and analysis of protein–protein interaction networks

To explore the interaction of DEGs, we submitted the DEGs to the STRING database(http://string.embl.de/), and only validated interactions with a combined score >0.4 were selected as significant. Cytoscape software was used to visualize and analyze the PPI network. The top 20 MCC genes were calculated and identified by CytoHubba as preselected coexpressed hub genes of SS and HT.

### 2.4. Patients and clinical minor salivary gland biopsy samples

To validate the expression of hub genes, 24 salivary gland biopsy samples, including 9 SS with HT patients, 9 SS without HT patients, and 6 non-SS patients (Table 1), were obtained from patients admitted to The Second Affiliated Hospital, Zhejiang Chinese Medical University, Hangzhou, Zhejiang Province, China. SS patients were diagnosed by the 2016 American College of Rheumatology/European League Against Rheumatism classification criteria for pSS.^[10]^ The diagnosis of HT mainly depends on the positive of serum TPO-Ab, TG-Ab, and the diffuse heterogeneous changes or hypoechoic changes of thyroid ultrasound.^[11]^ The use of patient information and MSGB was sanctioned by the Ethics Committee of The Second Affiliated Hospital, Zhejiang Chinese Medical University (Ethics Approval No. 2020-KL-011–01).

**Table 1:** Patients clinical and serological information.

Type	SS with HT	SS	Non-SS	*P*
Age (years)	54 ± 8.75	49 ± 16.90	55 ± 9.37	0.67
Gender (% female)	8 (88.89)	9 (100)	6 (100)	0.43
FS+ (%)	9 (100)	7 (77.78)	0 (0)	< 0.001
ANA+ (%)	7 (77.78)	8 (88.89)	3 (50)	0.24
Anti-SSA+ (%)	7 (77.78)	7 (77.78)	0 (0)	0.005
Anti-SSB+ (%)	2 (22.22)	2 (22.22)	0 (0)	0.465
TG-Ab	286.54 ± 410.38	1.53 ± 1.05	1.32 ± 0.90	0.047
TPO-Ab	224.99 ± 378.09	0.42 ± 0.42	0.17 ± 0.19	0.079

ANA+ = antinuclear antibody positive, Anti-SSA+ = Anti-Sjögren’s Syndrome A antibody positive, Anti-SSB+ = Anti-Sjögren’s Syndrome B antibody positive, FS+ = focus score ≥1, TG-Ab = thyroglobulin antibody., TPO-Ab = thyroid peroxidase antibody.

### 2.5. Immunohistochemical staining and validation of common hub genes

The main antibodies used for IHC were as follows: A PTPRC Rabbit IgG monoclonal antibody (ab40763), CD69 Rabbit IgG monoclonal antibody (ab233396) both from Abcam (Cambridge, 1:100; UK), and IKZF1 Rabbit IgG Polyclonal antibody (12016-A-AP), and LCP2 Rabbit IgG polyclonal antibody (12728-A-AP) both from Proteintech (Wuhan Sanying, 1:50; P.R.C.). Salivary gland samples were fixed in 10% formalin, embedded in paraffin blocks, and processed as continuous sections (4 µm thick). They were rehydrated through an alcohol gradient and stained with H&E. They were processed for antigen retrieval with EDTA Antigen Retrieval solution (pH 8.0) before blocking endogenous peroxidase activity with 3% H_2_O_2_ for 15 minutes. After incubation with PTPRC, CD69, IKZF1, and LCP2 antibodies overnight at 4°C, the sections were exposed to goat antirabbit antibodies at 37°C for 60 minutes. To assess antigen expression, each section was photographed at ×200× magnification and 5 images were taken over the entire section. Based on the staining characteristics of the immunohistochemical biomarkers, overall, nuclear and membrane expression were analyzed on Aperio ImageScope (Leica Biosystems, Germany). Positive pixel count v9 algorithm of Aperio ImageScope software was used for analysis (positive rate of immunohistochemical results = number of positives + number of strong positives)/total area). To further determine the prediction values of the positive expression rate of 4 genes for SS, ROC curves were constructed among the 3 groups. The plot area under the curve (AUC) was calculated through numerical integration of the ROC curves. The genes with the highest AUC values were the most diagnostic for SS.

### 2.6. Immune cell infiltration analysis using CIBERSORTx

CIBERSORTx (https://cibersortx.stanford.edu/) was used to analyze the differences in immune infiltration between SS and HT groups to evaluate the role of immune microenvironment in SS and HT formation. The results were then visualized using box-line plots.

### 2.7. Statistical analysis

Numerical results were presented as the means ± standard deviations or medians with interquartile ranges (IQR). Possible differences in demographic, clinical variables, and positive expression rate among the groups were evaluated by one-way analysis of variance (ANOVA) from more than 2 groups. Differences between the 2 groups were analyzed using an unpaired t-test with Welch correction. All analyses were performed using GraphPad Prism version 8.0.2 (GraphPad Software, San Diego, CA, USA) and statistical software package SPSS (version 22.0, SPSS Inc., Chicago, IL, USA). *P* < .05 was considered statistically significant.

## 3. Results

### 3.1. Identification of DEGs

**Figure 1.**
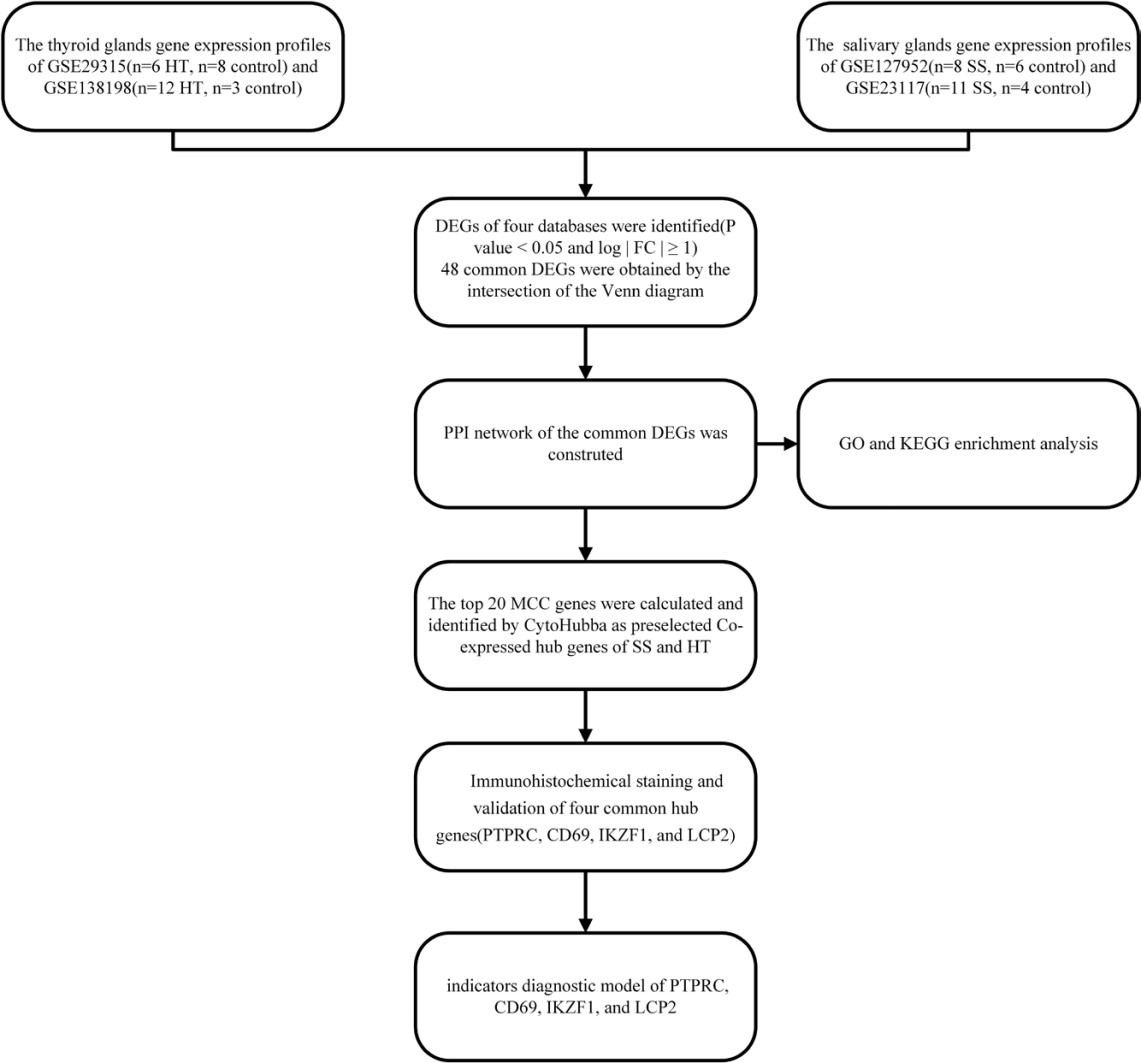
Research design flowchart.

**Figure 2.**
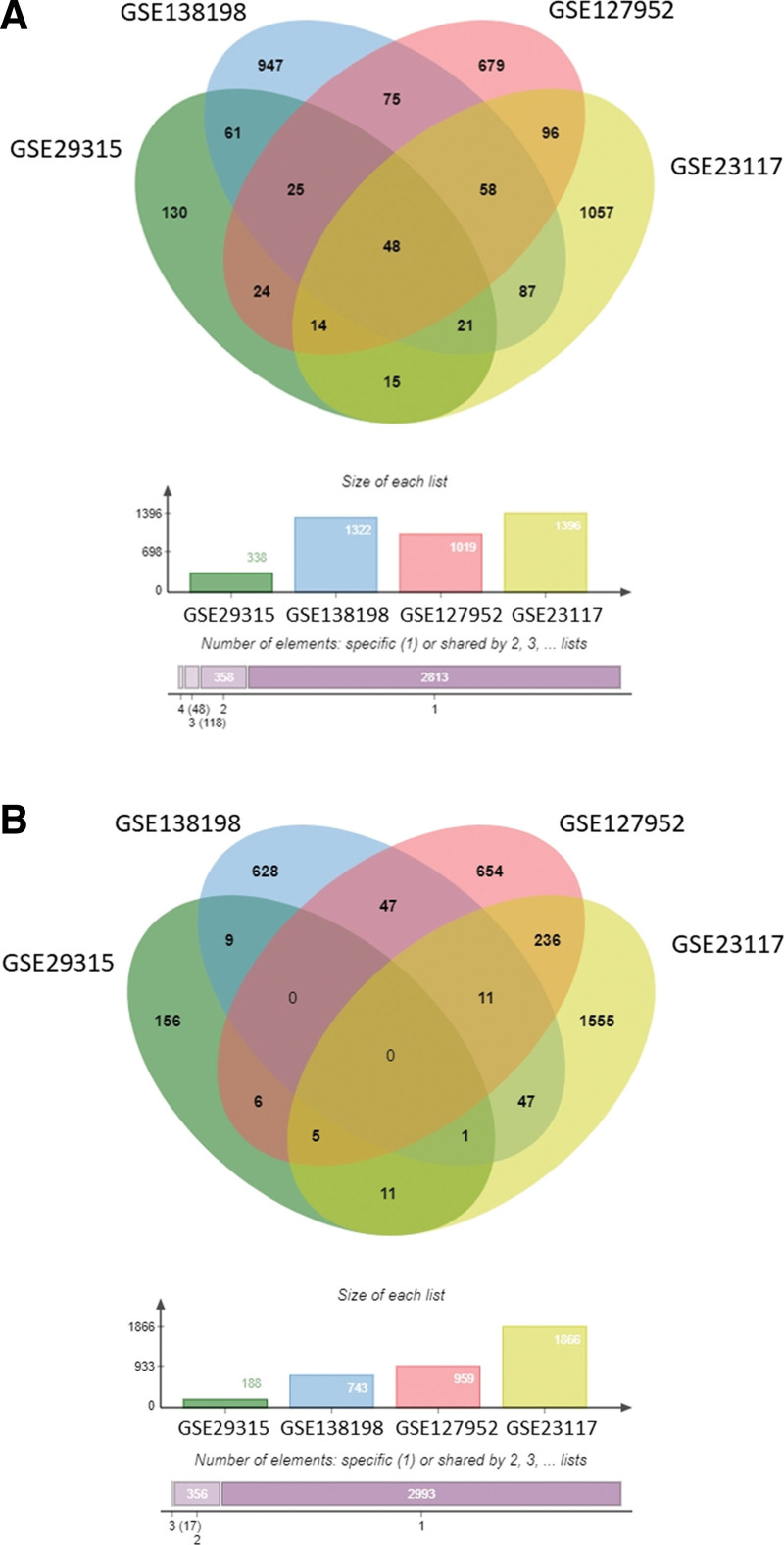
Venn diagrams show the DEGs in the salivary glands of patients with SS. Venn diagrams showing the upregulated DEGs (A) and downregulated DEGs (B). DEGs = differentially expressed genes.

The research flow chart of this study is shown in Figure 1. Four GEO datasets, which included SS patients, HT patients, and healthy controls, were analyzed with Venn tool. From the GSE29315, GSE138198, GSE127952, and GSE23117 datasets, we identified 48 upregulated genes that did not share a common downregulated gene (Fig. 2).

### 3.2. ClueGO enrichment analysis

**Figure 3.**
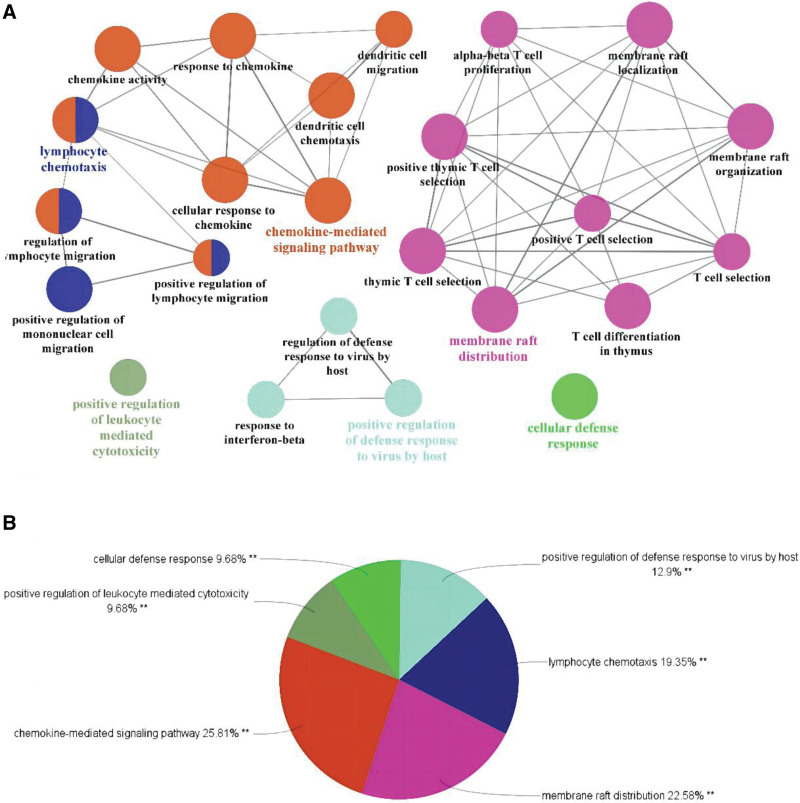
ClueGO enrichment analysis. Significantly enriched GO terms of DEGs based on GlueGO (biological processes) and CluePedia functions (A). The pie chart of ClueGO enrichment analysis (B). DEGs = differentially expressed genes, GO = gene ontology.

Enrichment analysis was performed on 48 cross-targets using the ClueGO v2.5.7 plugin. After setting the *P* value as < 0.05, Kappa Score Threshold as 0.4, and Go level as 3Minimum#Genes and 5%#Genes, we identified 24 GO terms (Fig. 3A). The upregulated DEGs were basically enriched in positive regulation of defense response to virus by host, membrane raft distribution, positive regulation of lymphocyte-mediated cytotoxicity, cellular defense response, and chemokine-mediated signaling pathway (Fig. 3B).

### 3.3. The Kyoto Encyclopedia of Genes and Genomes pathway enrichment analysis of DEGs

**Figure 4.**
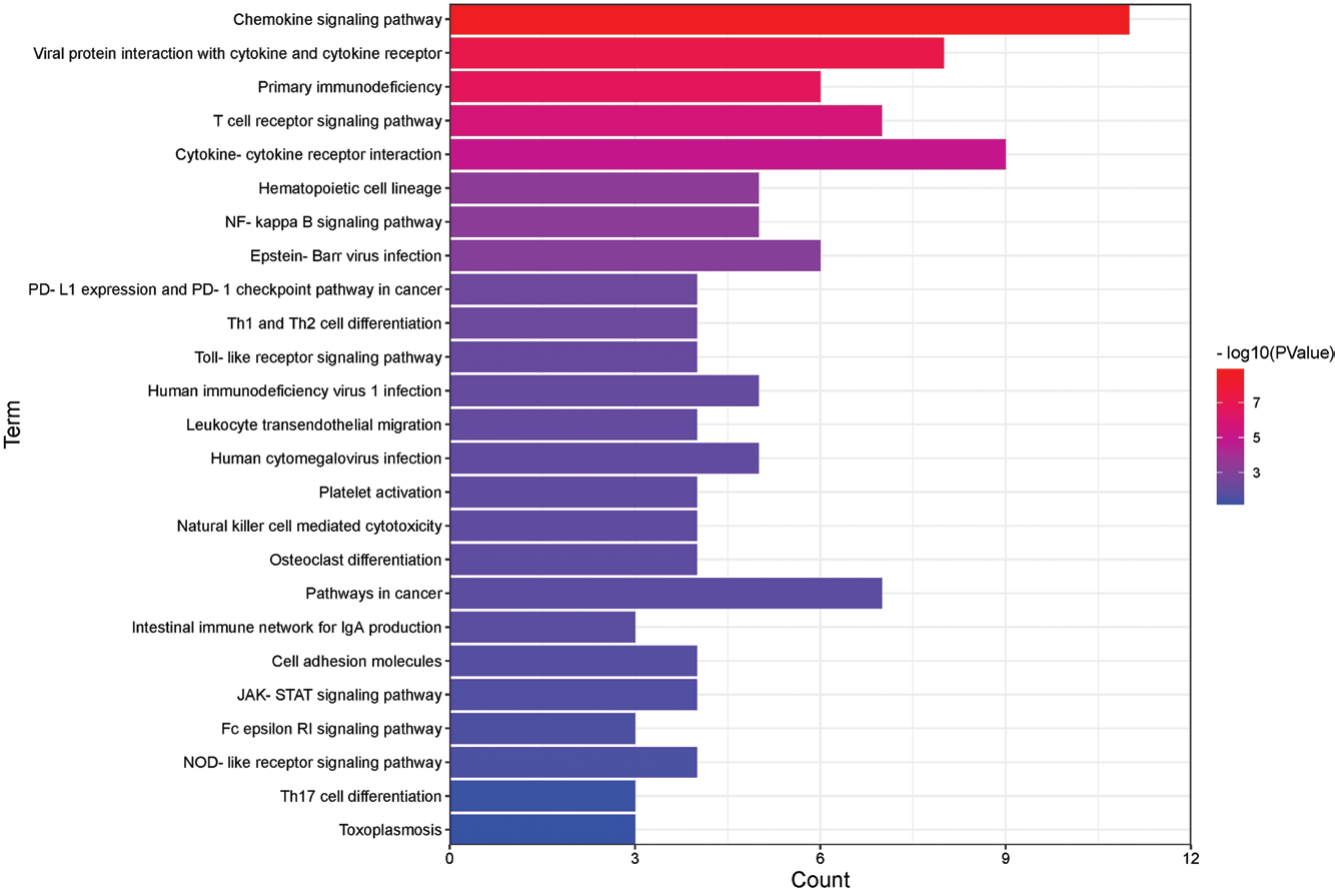
KEGG pathway terms of DEGs. A histogram of KEGG enrichment analysis of upregulated genes, which shows a gradual change in color from red to blue, indicating the change in the *P* value from small to large. DEGs = differentially expressed genes, KEGG = Kyoto Encyclopedia of Genes and Genomes.

The KEGG pathway enrichment analysis identified 27 significant pathways that included 48 upregulated genes. The 5 most significantly enriched pathways were chemokine signaling pathway, viral protein interaction with cytokine and cytokine receptor, primary immunodeficiency, T-cell receptor signaling pathway, and cytokine-cytokine receptor interaction (Fig. 4).

### 3.4. Construction of PPI networks of the upregulated genes and top 20 hub genes

**Figure 5.**
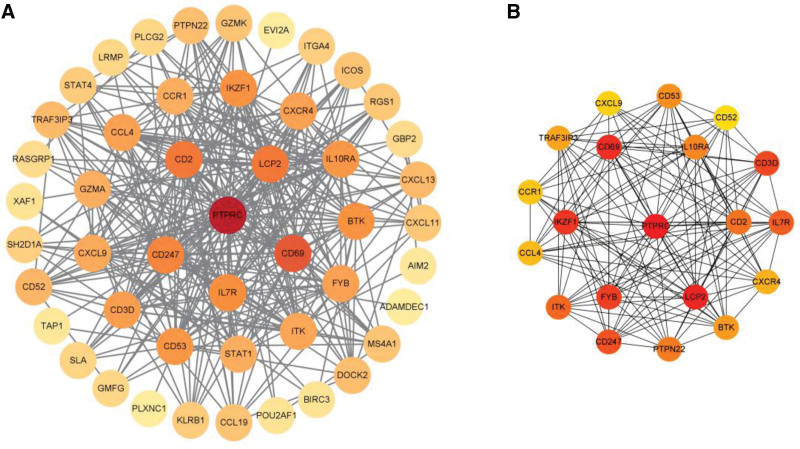
PPI networks for 48 upregulated DEGs (A) and the top 20 hub genes (B) were constructed using Cytoscape visualization, which shows a gradual change in color from yellow to red, indicating the change score from small to large. DEGs = differentially expressed genes, PPI = protein–protein interaction.

PPI networks of the DEGs were established using STRING database and Cytoscape. After removing 3 loose nodes, the PPI network contained a total of 48 nodes and 315 edges. We found the top 20 hub genes, including PTPRC, LCP2, CD69, IKZF1, FYB, CD3D, CD247, IL7R, ITK, CD2, PTPN22, IL10RA, CD53, BTK, TRAF3IP3, CXCR4, CCL4, CCR1, CXCL9, and CD52 utilizing Cytohubba based on MCC. We identified that the degree values were largest for receptor-type tyrosine-protein phosphatase C (PTPRC), lymphocyte cytosolic protein 2 (LCP2), cluster of differentiation 69 (CD69), Ikaros family zinc finger 1 (IKZF1), FYN binding protein (FYB) (Fig. 5).

### 3.5. Immunohistochemical validation of PTPRC, LCP2, CD69, and IKZF1 as the hub genes

**Figure 6.**
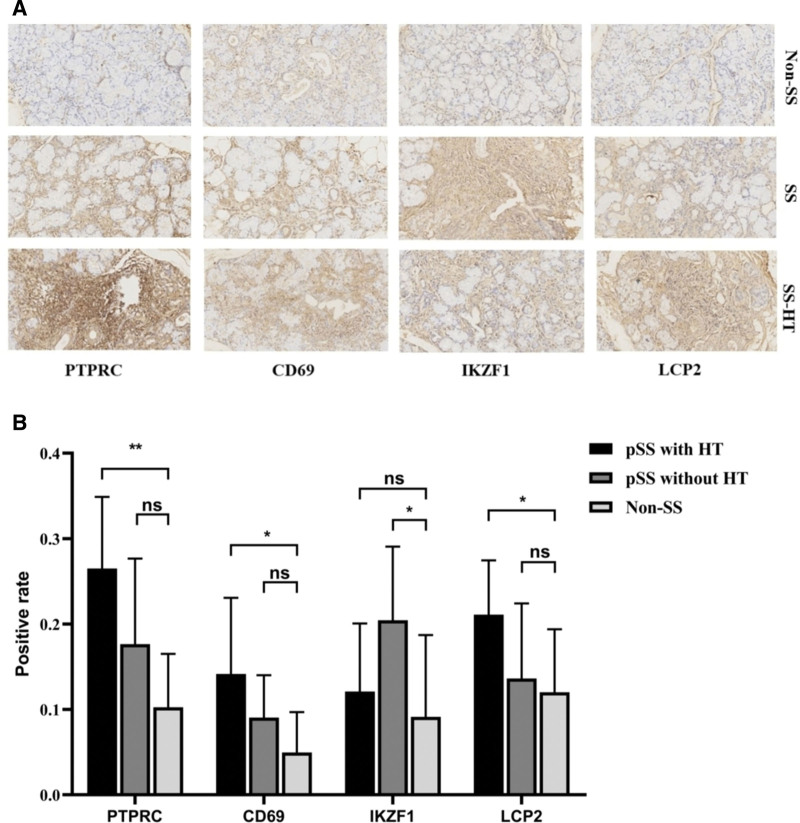
H&E and immunohistochemical analysis of PTPRC, CD69, IKZF1, and LCP2 proteins in minor salivary gland biopsy (MSGB) staining from 9 SS with HT patients, 9 SS without HT patients, and 6 non-SS patients(magnification: ×200 for H&E staining and × 200 for IHC) (A). Blue marks the nucleus, and brown marks the target protein. The bar graph indicated medians within the quartile ranges of positive rate (B). ns, not significant, *, *P* < .05; **, *P* < .01.

We selected MSGB samples from 9 SS with HT patients, 9 SS without HT patients, and 6 non-SS patients to verify the expression level of the PTPRC, LCP2, CD69, and IKZF1 by H&E and immunohistochemistry. Compared with non-SS patients, the expression of PTPRC, CD69, and LCP2 protein in the salivary gland of SS with HT group was significantly different from that of non-SS group, but there was no significant difference between SS without HT group and non-SS group; The expression of IKZF1 protein in SS without HT group was significantly higher than that in non-SS group, but there was no significant difference between SS with HT group and non-SS group (Fig. 6).

### 3.6. Diagnostic discrimination of the selected hub genes

**Figure 7.**
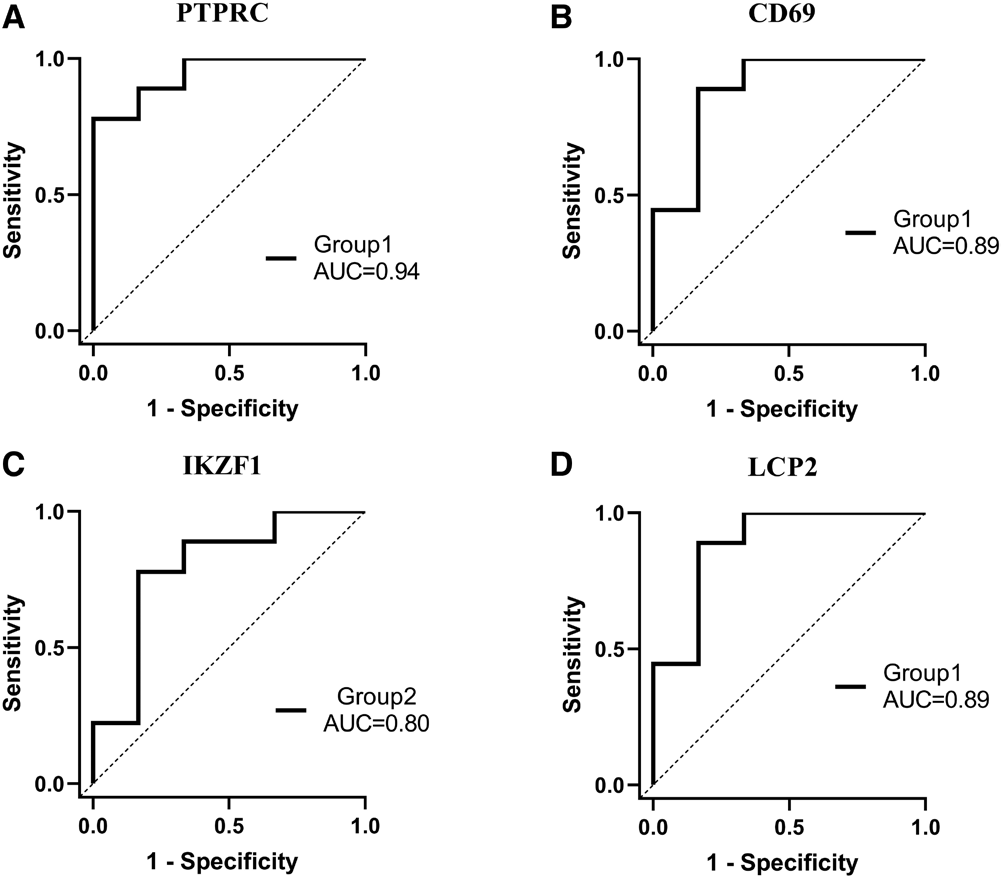
ROC curves analysis of positive rate of immunohistochemical results for 3 groups (Group 1: SS with HT patients, Group 2: SS without HT patients). (A) PTPRC, (B) CD69, (C) IKZF1, (D) LCP2. An AUC > 0.8 indicated that the predicted model had good efficacy. The AUC was also shown. AUC = area under the curve, ROC = receiver operating characteristic.

The sensitivity and specificity of these 4 hub genes as potential diagnostic biomarkers in SS patients were investigated by ROC analysis. The AUC of PTPRC in group 1 (SS with HT patients) was 0.9444 (*P* = .0047) (Fig. 7A). The AUC of CD69 in group 1 was 0.8889 (*P* = .013) (Fig. 7B). The AUC of IKZF1 in group 2 (SS without HT patients) was 0.7963 (*P* = .0593) (Fig. 7C). The AUC of LCP2 in group 1 was 0.8889 (*P* = .0133) (Fig. 7D). PTPRC, CD69, and LCP2 were shown to be able to well differentiate SS patients from non-SS.

### 3.7. Immune cell infiltration analysis by CIBERSORTx

**Figure 8.**
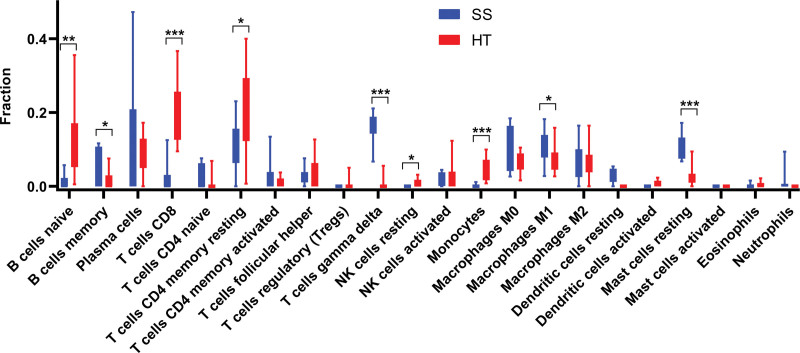
Results of immune infiltration by CIBERSORTx. Box-plot presented the difference of immune infiltration of 22 kinds of immune cells. *P < .05; **P < .01; ***P < .001.

CIBERSORTx was used to identify the types of immune cells involved in the formation of SS and HT. We obtained the proprotions of 22 kinds of immune cell types between SS and HT. SS Compared with HT, the proportions of B cells memory, T cells gamma delta, macrophages M1, and mast cells resting were relatively high, while B cells naive, T cells CD8, T cells CD4 memory, T cells CD4 memory resting, monocytes, and mast cells activated were low (Fig. 8).

## 4. Discussion

More and more research reports have shown that patients with SS had a higher risk of HT than those without SS. A monocentric retrospective study revealed overt thyroid poly autoimmunity in 15.7% of patients with SS.^[8]^ A large number of information have emphasized that HT and SS can be considered pathogenetically associated. It has been reported that thyroid and epithelial cells expressed the same human leukocyte antigen (HLA) molecules class II: HLA-B8 and HLA-DR3, and the tissue infiltration of the 2 disorders is mainly composed of CD4+T lymphocytes, and may formation of germinal center-like structures.^[12]^

Up to now, the contact mechanism between SS and HT has not been fully understood. Therefore, it is of great clinical significance to explore the molecular mechanism, early recognition, and intervention between SS and HT. This study combined bioinformatics and salivary gland validation to discover the possible copathogenesis mechanism of SS and HT. The expression of PTPRC, CD69, and LCP2 in SS patients with HT was significantly higher than that in the control group, and there was no significant difference between the expression of the above genes in SS patients without HT and that in the control group, which suggested that the above genes might be the key way of their copathogenesis, and further verified the correlation between SS and HT at the genetic level. We used cibersortX to investigate the differences in immune cell infiltration in the salivary gland of SS versus the thyroid of HT and showed that they differed significantly between B cells, CD4 T cells, and CD8 T cells, suggesting a degree of immune overlap between SS and HT patients. In this study, the pathogenic association between SS and HT was explored. Four hub genes were identified, and joint mechanisms were revealed for the co-occurrence of SS and HT.

Protein tyrosine phosphatase receptor C (PTPRC), also known as CD45, is a transmembrane glycoprotein that is a crucial regulator of antigen receptor-mediated activation of T and B cells.^[13]^ It accounted for about 10% of the surface antigens of T and B lymphocytes.^[14]^ PTPRC controlled immune function mainly by regulating cytokine responses and T-cell receptor (TCR) signaling, and the TCR signaling has been shown to be important in autoimmune disease pathogenesis.^[15]^ PTPRC inhibited the Janus kinase (JAK) and negatively regulates the receptor signaling pathway of cytokines, which in turn activates signal transducer and activator of transcription (STAT) family transcription factors.^[16]^ This suggests that PTPRC may be involved in the upstream regulation of the JAK-STAT pathway. JAK-STAT signal pathway is a signal transduction pathway that mediates the activation of various cytokines. It can lead to cell activation, proliferation, and the release of inflammatory factors, and can contribute to the differentiation of Th1 and Th17 cells. At present, the imbalance of this pathway has been found in many autoimmune diseases.^[17]^ Recently, it has been found that JAK inhibitors can alleviate the activation of salivary gland epithelial cells, thereby relieving inflammation and lymphocyte infiltration, and improving dryness symptoms.^[18]^ The regulation of cytokine responses is also a function of PTPRC. CD45RChigh and CD45RClow T cells produced interleukin-2 (IL-2), IL-4, IL-10, and IL-13.^[19]^ Phenekos et al reported that the proinflammatory cytokines IL-2, IL-4, and IL-10 were distinctly raised in HT.^[20]^ This suggests that proinflammatory cytokines may be a significant pathway for CD45 to influence the pathogenesis of HT.

CD69 belongs to the C-type lectin receptor family and is one of the early markers of T lymphocyte activation. It participated in a variety of cellular reactions in vivo and further enhances T-cell proliferation as a co-stimulatory signal. Moreover, CD69 promoted the expression of various cytokines and the synthesis, secretion, and expression of inflammatory mediators.^[21]^ One of the important mechanisms by which CD69 exerted its immunomodulatory effect was to control the activity of Th17 and Treg.^[22]^ CD69 expression inhibited Treg activity by upregulating transforming growth factor-β (TGF-β) in systemic sclerosis.^[23]^ Miao et al reported that the number of Th17 cells and Treg cells in the salivary glands of pSS were positively correlated with the score of inflammation.^[24]^ Therefore, we speculate that CD69 may lead to SS by affecting Th17/ Treg balance. In HT, CD69 cells with high expression in infiltrating T cells can activate the expression of first apoptosis signal ligand (FasL), and then destroyed thyroid cells.^[25]^ As a marker of cell activation, CD69 may be able to judge the disease activity of SS and HT by detecting the expression of CD69 on the surface of T cells.

Ikaros family zinc finger 1 (IKZF1) is one of the most significant transcription factors that control the specificity and differentiation of lymphocytes. It regulated lymphocyte differentiation, proliferation, and self-tolerance through regulation of B-cell, T-cell, and dendritic cell signaling, which were important to maintain the immune system stability and avoid the occurrence of autoimmune diseases.^[26–28]^ Furthermore, previous studies have shown that IKZF1 is involved in the regulation of STAT4 in human T cells.^[29]^ Additionally, interferon regulatory factors 5 can affect the induction of inflammatory cytokines and interferon by regulating the expression of IKZF1.^[30,31]^ Moreover, the correlation of pSS with interferon regulatory factors 5 and STAT4 has been demonstrated in GWAS.^[32]^ Our experimental evidence further confirmed that IKZF1 is related to pSS, and it is a highly specific and sensitive biomarker. IKZF1, IRFs, and STAT4 may play a role in the pathogenesis of diseases by regulating immune-related cell activities involved in some signal pathways of pSS, such as interferon pathway. In contrast, IKZF1 has not been studied in HT, and there is no significant difference between SS with HT group and the control group in the expression of salivary gland immunohistochemistry.

Lymphocyte cytosolic protein 2 is an adapter protein-encoding gene necessary for the normal development of T cells and activation of serine phosphorylation and participates in the protein tyrosine kinase pathway activated by the TCR.^[33,34]^ Although there are no LCP2-related studies in HT and SS, studies in other medical fields detail the function of LCP2 in immune cells. The function of LCP2 protein in T cell differentiation and activation has been reported. LCP2 protein regulated Th1 and Th2 cell function through serine phosphorylation, which was essential for T cell differentiation.^[34]^ LCP2 played an important role in NK cell-mediated recognition of missing self-targets^[35]^ and actively regulated antigen-induced mast cell activation by recruiting B-cell receptor (BCR).^[36]^ These studies indicated that LCP2 was deeply involved in the immune response by regulating immune cells. The higher expression of LCP2 in the salivary gland of SS with HT group suggests that abnormal expression of LCP2 may be a factor in the development of the disease, which needs to be further investigated.

## 5. Conclusion

Based on the existing transcriptome data, this study provided new insights into the common pathogenesis of SS and HT. PTPRC, LCP2, and CD69 were established as pivotal genes involved in the development of SS combined with HT and integrated into the diagnostic model of SS. This may also be the genetic and molecular basis for the clinical comorbidity of SS with HT. To our knowledge, this is the first study to explore and verify the common pathogenesis of SS with HT using quantitative data of IHC. The exploration of SS combined with HT at the molecular mechanism level will help us to further understand the mechanism of abnormal inflammatory reactions and immune dysfunction in the pathogenesis of these 2 diseases. This study put forward some new ideas into the shared pathogenesis and mechanisms of the occurrence of SS and HT. Moreover, the main limitation of this study was the small sample size and the lack of genetic validation in thyroid tissue samples because of the difficulty of obtaining thyroid tissue from patients and healthy individuals.

## Acknowledgments

We are indebted to all the subjects who took part in this study, and the staff of the Department of Rheumatology, The Second Affiliated Hospital, Zhejiang Chinese Medical University.

## Author contributions

**Conceptualization:** Liying Chen, Xinchang Wang.

**Data curation:** Kaiyuan Zhang, Xue Yu, Xinchang Wang.

**Formal analysis:** Xue Yu, Tao Hong.

**Investigation:** Xue Yu, Dingqi Lu.

**Methodology:** Yunxin Zhang, Dingqi Lu, Yating Ren.

**Resources:** Xinchang Wang.

**Software:** Xinyi Yao, Liying Chen.

**Validation:** Kaiyuan Zhang, Yunxin Zhang, Xinyi Yao.

**Writing – original draft:** Kaiyuan Zhang, Yunxin Zhang, Xinchang Wang.

**Writing – review & editing:** Tao Hong, Yating Ren.
